# Trop2 expression, p16 expression status, and histologic subtype in carcinoma of the uterine cervix

**DOI:** 10.3389/pore.2025.1612252

**Published:** 2025-11-07

**Authors:** Grit Gesine Ruth Hiller, Benjamin Wolf, Mirjam Forberger, Annekathrin Freude, Christine Elisabeth Brambs, Svenja Droste, Lars-Christian Horn, Anne Kathrin Höhn

**Affiliations:** 1 Division of Gynecologic, Breast and Perinatal Pathology, Institute of Pathology, University Hospital Leipzig, Leipzig, Germany; 2 Division of Gynecologic Oncology, Department of Obstetrics and Gynecology (Institute of Trier), University Hospital Leipzig, Leipzig, Germany; 3 Department of Obstetrics and Gynecology, Cantonal Hospital Lucerne, Lucerne, Switzerland

**Keywords:** cervix, squamous cell carcinoma, adenocarcinoma, Trop2, targeted therapy

## Abstract

**Introduction:**

The antibody-drug conjugate (ADC) Sacituzumab-Govitecan (SG), a humanized anti-Trop2 monoclonal antibody linked to the cytotoxic topoisomerase I inhibitor SN38, achieved promising results in the treatment of various solid tumors. Treatment approaches with SG requires the expression of Trop2 within tumor cells. The present study explored immunohistochemical Trop2 expression in cervical carcinomas in correlation with histologic subtypes and p16 expression status.

**Material and methods:**

Using an immunoreactive score (IRS), immunohistochemical Trop2 expression in surgically treated cervical carcinoma specimens was evaluated by comparing squamous cell carcinomas and adenocarcinomas, and the expression status of p16 as a surrogate marker for high-risk HPV infection.

**Results:**

A total of 101 cases were included in this study. Of these 75% were squamous cell carcinomas, and 25% were adenocarcinomas, and 5% showed negative immunoexpression for p16, indicating HPV-independent carcinoma. All tumors showed at least weak Trop2 expression. There were no differences in the mean Trop2 IRS-scores comparing histological subtype [squamous: 8.5 (3–9) vs. adeno: 6 (1–9); p = 0.8] and p16 expression status [positive: 9 (6–9) vs. negative: 8 (6–9; p = 0.6]. No differences in Trop2 expression were observed when post-surgical tumor stage, pelvic lymph node status and peritumoral stromal remodelling (desmoplastic response and peritumoral infiltrating lymphocytes) were analysed.

**Conclusion:**

Regardless of the histologic tumor type and p16 expression status, cervical carcinomas show high Trop2 expression and, therefore, may represent a promising therapeutic target. Clinical trials exploring Trop2 directed ADCs such as Sacituzumab-Govitecan are warranted in this cancer type, including the prognostically poor HPV-independent (p16 negative) tumors, mainly adenocarcinomas.

**Significance:**

Regardless of the histologic tumor type and p16-expression status, cervical carcinomas show high Trop2 expression, which may therefore represent a promising therapeutic target in these tumors.

## Introduction

Cervical carcinoma (CX) is the 8th most common malignancy with yearly 661.021 newly diagnosed cases worldwide and 348.189 cancer related deaths [1]. The vast majority of cases are squamous cell carcinomas (CSCC), and approximately 25% represent endocervical adenocarcinomas (EAC) [2–4].

The 5-year overall survival rate for all stages of cervical carcinoma is 67% [5]. Regardless of the treatment approach, approximately 25% of patients with FIGO stage > IIB will experience recurrence [6, 7] with a consecutive limited overall survival of 35.9% [4]. Irrespective of the histological subtype, clinicopathological features in surgically treated patients, such as tumor stage, inguinal lymph node involvement, tumor size and margin status, are associated with the prognosis of CX [8, 9].

Recent data have shown a prognostic impact of HPV status, mainly in tumors with adenocarcinomatous histology [10, 11]. Despite new developments in the treatment of CX [12–16], therapeutic options for locally advanced and recurrent disease are limited [7, 17] and to our knowledge, there have been no studies on the association between Trop2 and p16 expression to date [18].

Trop2 (trophoblast cell surface antigen 2) was first described in 1981, showing that it is highly expressed in the human placental trophoblastic cells [19]. Under physiological conditions, it plays an active role in regulating the stem cell proliferation, migration, and tissue regeneration [20, 21]. In cancer cells, Trop2 is involved in epithelial-mesenchymal transition, tumor cell proliferation, adhesion, and migration [20, 22, 23]. Trop2 overexpression has been reported in different types of carcinomas [20, 24] and is expressed in squamous cell carcinomas of various organs, including the head and neck [24–26], vulva [27, 28], and uterine cervix [24, 29, 30]. It is also expressed in tumors with adenocarcinomatous histology [23, 29, 31]. Interestingly, Trop2 overexpression in tumors seems to be modulated by a network of several transcription factors, and is not a result from gene amplification or mutations itself. However these processes are not yet fully understood [23, 32, 33].

Trop2 has already been established as a new target in cancer precision medicine, with multiple ongoing clinical trials [34]. The antibody-drug-conjugate (ADC) Sacituzumab-Govitecan (SG) was approved by the U.S. Food and Drug Administration (FDA) for the treatment of unresectable metastatic triple-negative breast cancer with two or more previous treatments based on the results of the phase III ASCENT trial [35]. SG contains a humanized anti-Trop2 monoclonal antibody and the topoisomerase I inhibitor drug SN-38 [36] and may play a role in the treatment of solid tumors beyond breast cancer [21, 23, 37].

For SG therapy to be effective, Trop2 must be expressed within cancer cells. Detailed data of Trop2 expression in cervical cancer are still limited, especially the association to p16 expression [3, 22, 24, 29, 30]. The present study was designed to evaluate the immunohistochemical expression for Trop2 in CX, with special emphasis on the histopathological tumor type (CSCC versus EAC) and its association with immunohistochemical p16 expression.

## Materials and methods

The study was approved by the Leipzig University Ethics Committee (151/2000, 192/2001, and 012/13–28012013; initial approval was granted on 22 September 2000, and the subsequent amendments were approved on 17 October 2007 and 6 March 2013). Consecutive surgical specimens from patients undergoing primary surgery (without any neoadjuvant therapy) using the total mesometrial resection (TMMR) technique developed by Höckel et al. [12] were extracted from the institutional archive of the Institute of Pathology at Leipzig University Hospital. The TMMR trial is registered at the University of Leipzig Cancer Centre (ULCC012-13-28012013).

### Peritumoral stromal remodelling

A desmoplastic stromal reaction (DSR) is a histological equivalent of peritumoral stromal remodelling. It results from dormant fibroblasts switching to myofibroblasts [38], classified as mature, intermediate, or immature [39].

Peritumoral infiltrating lymphocytes (pTIL) were evaluated using a three-category immunoscore analysis: a 0%–25% density was scored as low, a density between 25% and 75% was scored as intermediate, and a density between 75% and 100% was scored as high [40, 41].

DSR and pTIL were obtained from a microscopic field using a 10-fold objective at the front of invasion in one representative tumor slide.

### Immunohistochemical p16 expression

p16 immunostaining was performed in all cases using a mouse monoclonal antibody (Roche Cat# 805-4713, RRID:AB_3675558). p16 IHC in squamous cell carcinomas was interpreted as *positive* (i.e., overexpression) if there was continuous nuclear and cytoplasmic transepithelial ‘block-like’ staining and interpreted as *abnormal diffuse positive* in adenocarcinomas when staining showed a strong and diffuse positive expression nuclear or nuclear and cytoplasmatic, in accordance with the Lower Anogenital Squamous Terminology [42] and The British Association of Gynaecological Pathologists guidelines [43].

### Immunohistochemical Trop2 expression

**FIGURE 1:**
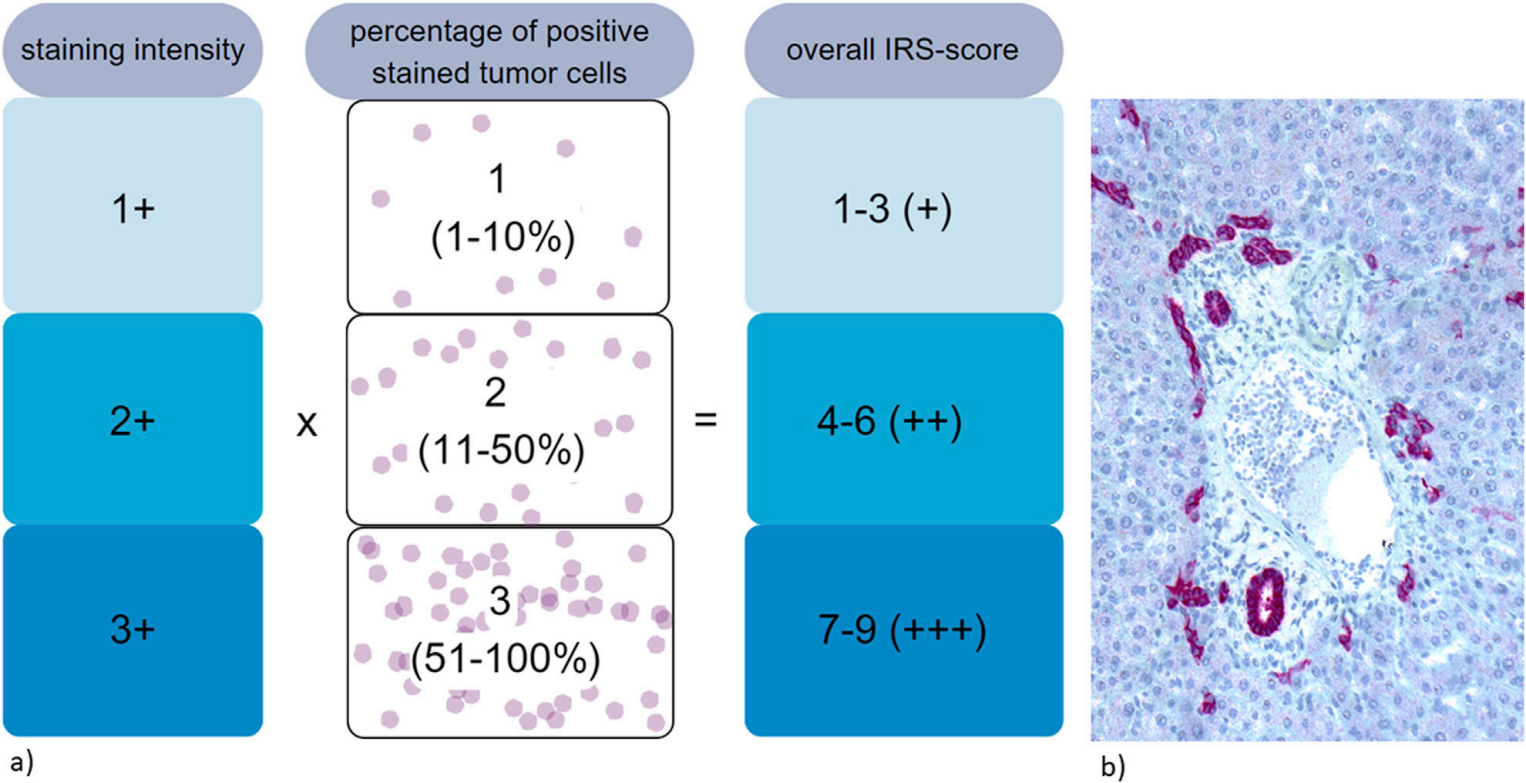
**(a)** Evaluation of immunohistochemical expression of Trop2 using immunoreactive score (IRS) [22, 28, 44]. **(b)** Strong and diffuse immunohistochemical Trop2 staining within intrahepatic bile ducts, used as positive control.

All slides were stained with a rabbit monoclonal antibody for Trop2 (Biozol Cat# MSV-3648-733R-1, RRID:AB_3676562). Trop2 expression was evaluated using an immunoreactive score (IRS) as previously described [22, 28, 44]. The staining intensity (SI) was scored as negative (0), weak (1), moderate (2), or strong (3). The percentage of positively stained tumor cells was calculated as follows: 0 (complete negative staining of tumor cells), 1 (1%–10% positive stained tumor cells), 2 (11%–50%), and 3 (51%–100%). The overall staining results were calculated as SI × percentage staining. A final score value of 0 was considered negative, scores of 1–3 as weak, scores of 4–6 as moderate, and scores of 7–9 as strong expression [22], see Figure 1A. Trop2 expression in intrahepatic bile ducts was used as positive control (Figure 1B).

The antibody details are summarised in Table 1.

**TABLE 1:** Immunohistochemical antibody information.

Antibody	Clone	Vendor	Dilution and pretreatment	Detection system
p16 (CINtec p16 Histology)	E6H4	Roche Diagnostics	ready to use CC1 36’/32′	DAB
Trop2 (TACSTD2)	MSVA-733R	MS validated antibodies/Biozol	1:150 CC1 20'/36′	FAST RED

Since Trop2 IRS correlates with the histopathologic subtype and p16 expression status, the evaluation of immunohistochemistry for Trop2 was performed by observers blinded to p16 expression status.

### Statistical evaluation

Data were organized in comma-separated value (CSV) spread sheets and analyzed using the statistical software R (R Core Team 2023). Continuous variables are presented as means or medians with standard deviation and range, respectively. Discrete data are presented as numbers and percentages. Fisher’s exact test and the chi-squared test were used to test for distributional differences between categorical variables, as appropriate, and the Mann-Whitney test was applied for continuous variables. Barplots were created using Excel 16.78 (Microsoft Corporation, United States, 2023).

## Results

**FIGURE 2:**
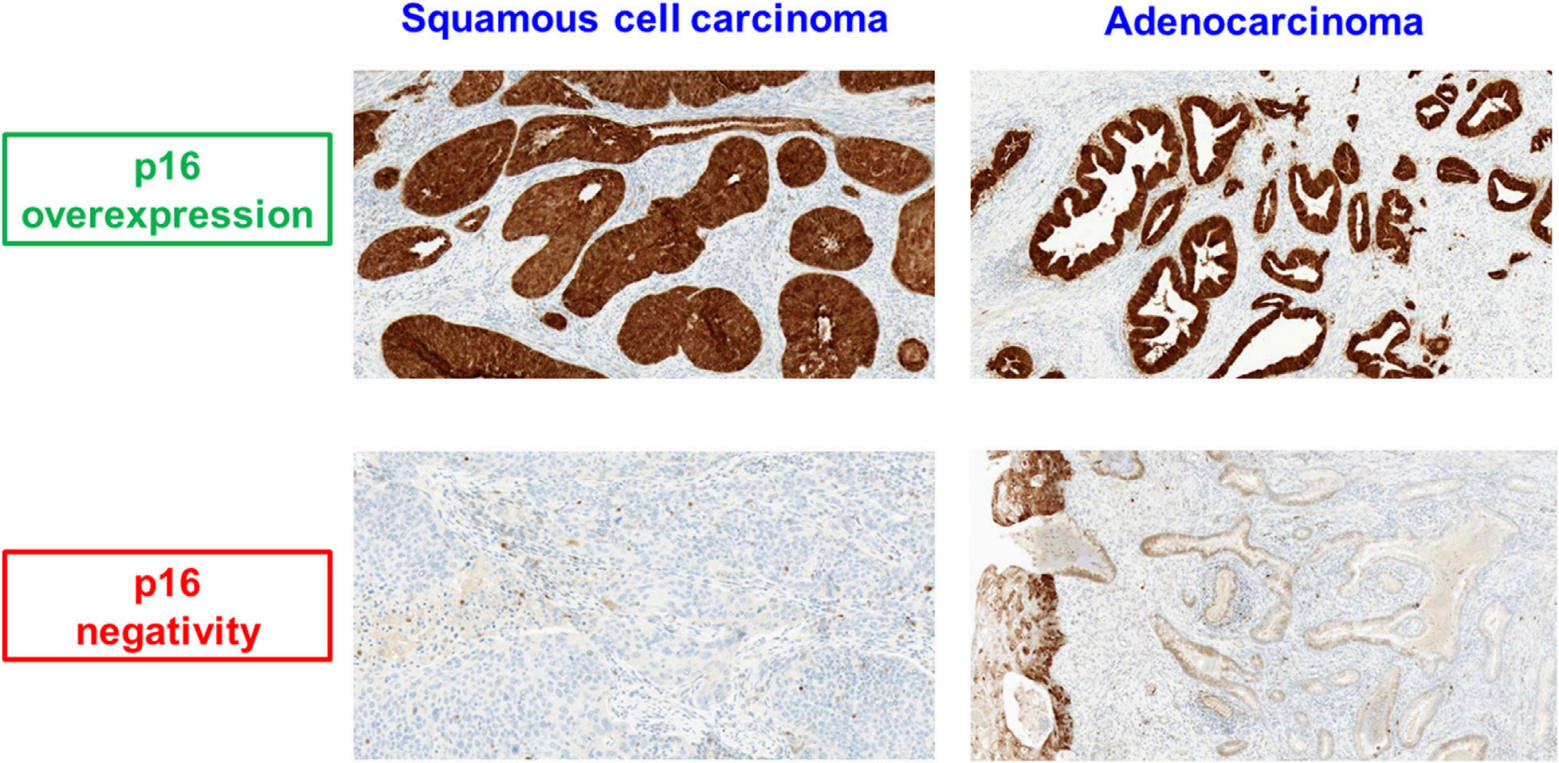
p16 expression in squamous cell carcinoma and adenocarcinoma of the uterine cervix.

A total of 101 patients were included in the analysis. The majority of cases were squamous cell carcinomas. Of all carcinomas 95% showed immunohistochemical p16-overexpression (Figure 2), indicating a high-risk HPV association. Patient characteristics are summarized in Table 2.

**TABLE 2:** Patient characteristics.

	All cases n = 101	Squamous cell carcinoma n = 76 (75%)	Adenocarcinoma n = 25 (25%)
Post-surgical stage (TNM 2017)			
pT1b1	43 (42.5%)	28 (36.8%)	15 (60%)
pT1b2	11 (10.9%)	8 (10.5%)	3 (12%)
pT2a	2 (2%)	1 (1.3%)	1 (4%)
pT2b	45 (44.6%)	39 (51.4%)	6 (24%)
Pelvic lymph node involvement			
No (pN0)	73 (72.3%)	54 (71%)	19 (76%)
Yes (pN1)	28 (27.7%)	22 (29%)	6 (24%)
p16 immunostaining			
No overexpression	5 (5%)	1 (1.3%)	4 (16%)
Overexpression	96 (95%)	75 (98.7%)	21 (84%)

All cases showed at least weak Trop2 expression within the tumor cells. The vast majority of all cervical carcinomas (96/101; 95%) showed moderate to strong Trop2 expression (IRS-score >4). Endocervical adenocarcinomas represented an insignificantly lower expression level of Trop2 compared to squamous cell carcinomas (adeno: 6 (1–9) vs. squamous: 8.5 (3–9); p = 0.8).

**FIGURE 3:**
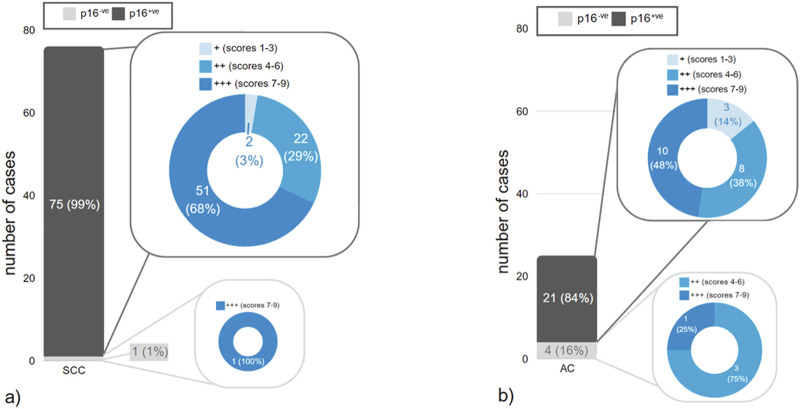
**(a,b)** Distribution of staining results for p16 within different histopathologic tumor types in correlation to different IRS-scores of Trop2 expression. **(a)** squamous cell carcinomas (SCC), **(b)** endocervical adenocarcinomas (EAC).

The median IRS for p16 overexpressing cases was 9 (range 6–9) versus 8 (range 6–9) in those without p16 overexpression (p = 0.6), indicating no differences in Trop2 expression depending on the p16 status in our cohort (see Figures 3A,B).

**FIGURE 4:**
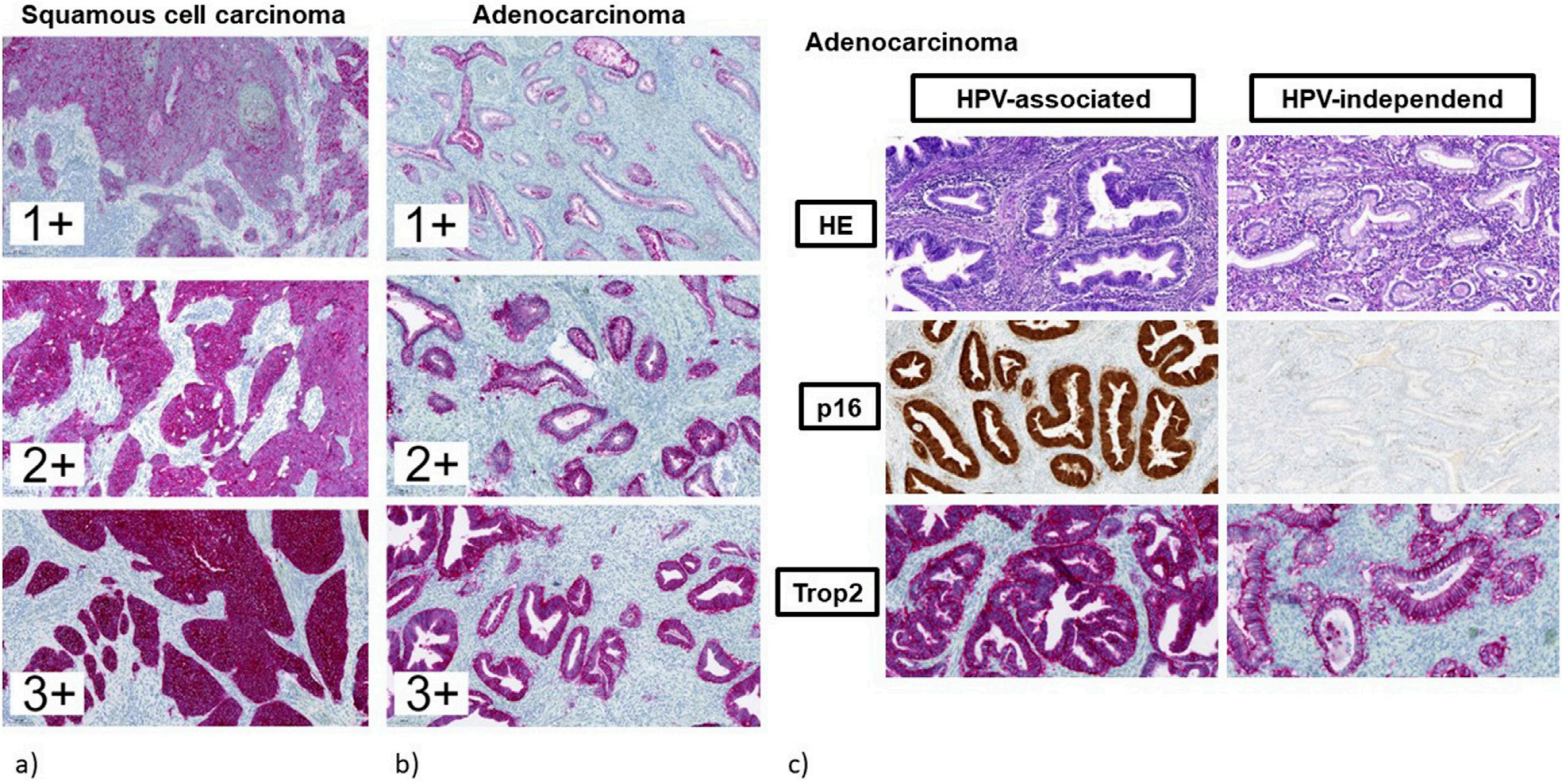
**(a–c)** Immunohistochemical staining for Trop2. **(a)** squamous cell carcinomas (regardless of p16 expression status). **(b)** endocervical adenocarcinomas (regardless of p16 expression status). **(c)** endocervical adenocarcinomas in correlation to p16 expression status. Left: adenocarcinoma of the usual type with abnormal diffuse positive expression of p16 indicating HPV-associated tumor with strong and diffuse Trop2 staining. Right: adenocarcinoma of the gastric type with negative expression of p16 indicating HPV-independent tumor with moderate Trop2 staining.

The different IRS for Trop2 in correlation with histological subtypes and p16 expression status are shown in Table 3. Representative Trop2 staining results are provided in Figures 4A–C.

**TABLE 3:** Trop2 expression in correlation to p16 expression status and histopathological subtype.

Trop2 expression	All cases n = 101	Squamous cell carcinoma n = 76 (75%)		Adenocarcinoma n = 25 (25%)	
		p16+ n = 75 (98.7%)	p16-n = 1 (1.3%)	p16adp^a^ n = 4 (16%)	p16neg^b^ n = 21 (84%) serous 35.7% mucinous 28.6% usual type 28.6% villoglandular 7.1%
Immunoreactive Score (IRS)					
Mean (range)	7.71 (1–9)	7.96 (3–9)	9 (9–9)	6.9 (1–9)	6.75 (6–9)
P-value		0.5		0.7	
IRS group					
Negative	0 (0%)	0 (0%)	0 (0%)	0 (0%)	0 (0%)
+ (scores 1–3)	5 (5%)	2 (2.7%)	0 (0%)	0 (0%)	3 (14.3%)
++ (scores 4–6)	33 (32.6%)	22 (29.3%)	0 (0%)	3 (75%)	8 (38.1%)
+++ (score 7–9)	63 (62.4%)	51 (68%)	1 (100%)	1 (25%)	10 (47.6%)
P-value		0.8		0.6	

^a^ abnormal diffuse positive.

^b^ negative/patchy.

Statistically significant differences were not observed regarding tumor stage, pelvic lymph node involvement, and parameters of peritumoral stromal remodelling when comparing Trop2 low (IRS-score <3) and Trop2 high (IRS-scores >7) cases (Table 4).

**TABLE 4:** Trop2 expression in correlation to clinicopathologic characteristics.

	Trop2 expression level		
	Low/moderate (IRS ≤8)	High (IRS 9)	p-value
Post-surgical tumor stage			
pT1b	18 (47%)	25 (40%)	0.4
≥ pT2a	20 (53%)	38 (60%)	
Pelvic lymph node involvement			
pN0	9 (24%)	19 (30%)	0.5
pN1	29 (76%)	44 (70%)	
Peritumoral infiltrating lymphocytes			
None/low	22 (58%)	33 (52%)	0.6
Intermediate/high	16 (42%)	30 (48%)	
Desmoplastic stromal response			
None	10 (26%)	24 (38%)	0.2
Present	28 (74%)	39 (62%)	

## Discussion

Cervical cancer patients with early stage disease show a satisfactory prognostic outcome after radical surgery and/or chemoradiation [13, 17]. Nevertheless, patients with locally advanced disease and HPV-independent tumors show reduced overall survival [4, 17, 45–47]. Despite promising results in immune checkpoint inhibition [14–16], the treatment of recurrent cervical cancer remains challenging, predominantly due to the lack of other targeted treatment options and limited response to traditional gynecological cancer chemotherapies. In some centers, pelvic exenteration may be an option [13, 48] but it is of limited success, especially in patients with previous pelvic radiation therapy [49]. For these patients there is an unmet clinical need for novel targeted treatment approaches that are both effective and have limited adverse side effects.

The use of ADC has opened a new window for targeted treatment of a variety of solid tumors and targets [20, 22, 33, 48]. In ADCs, an antibody that binds to a specific target on tumour cells is linked to a cytotoxic drug. The ADC Sacituzumab Govitecan (SG) consists of an anti-Trop2 antibody linked to the topoisomerase I inhibitor SN38 [22, 33, 48]. SG has been tested in various clinical trials with promising results in a variety of cancer entities [20, 22, 35, 48].

The expression of cellular targets (e.g., Trop2 for the treatment with SG) is required for the treatment approach with ADCs and several trials using Trop2-directed ADCs are progressing [34].

The FDA approved SG for urinary bladder cancer and locally advanced or metastatic breast carcinoma [50, 51].

Within the treatment approach for SG there is no need for predictive immunohistochemical testing [50]. However, previous ADC-directed trials have failed due to inappropriate patient selection for this treatment. In patients with ovarian cancer the FOREWARD I trial (a folate receptor-α (FR-α) targeted ADC Mirvetuximab Soravtansine) failed because all patients with immunohistochemical FR-α expressing tumors (regardless of staining intensity and number of positively stained cells) were treated [52]. The following SORAYA-trial represented promising results within this treatment, as only patients with FR-α overexpression (i.e., 75% of viable tumor cells exhibiting at least 2+ level FR- α) were treated [53], which led to the FDA approval of that ADC [54]. With regard to Trop2, the ASCENT trial has shown that patients with metastatic triple negative breast cancer have an improved overall survival rate when treated with SG if Trop2 is moderately to highly expressed [52]. So, the results of those three trials highlight the necessity to obtain immunohistochemical expression data for different targets within ADC treatment [55].

Interestingly, it is not possible to estimate the response to ADCs in all cases based solely on the IHC assessment or the target proteins. For example, Tisotumab-Vedotin (TV) represents another FDA-approved ADC for the treatment of pre-treated recurrent and/or metastatic cervical cancer [53]. In TV, an antibody directed against tissue factor is linked to the microtubule disrupting agent monomethyl-auristatin E. Tissue factor acting as the targeted antigen in TV is expressed within the cell membrane of cervical cancer tissue samples within this study [53, 54]. In an exploratory analysis, different expression status in tumor biopsy samples from 374 cervical cancer patients was compared with overall response to TV [54] and showed no correlation between membranous expression status of tissue factor and treatment outcome.

The present study evaluated Trop2 expression in cervical cancer and showed that almost all cancers were positive for Trop2, regardless of histological tumor type (squamous versus adenocarcinomas) and p16 expression status (Table 3; Figures 3A,B, 4A–C). When comparing normal cervical tissue, cervical intraepithelial neoplasia (CIN), and invasive cancers, Liu et al. [22] reported an increase in Trop2-positive cases (normal cervical tissue 45%; CIN 64.5%; carcinomas 88.7%; p < 0.001). Within CIN-lesions there was a gradual increase in Trop2 expression from CIN 1 (50%) to CIN 2 (66.7%) and CIN 3 (76.9%; p = 0.037). All the tumors in the present study showed immunohistochemical Trop2 expression. The overall reported Trop2 positivity in cervical carcinoma ranges from 84.6% to 98.5% [3, 22, 29, 30].

In the present study, the mean IRS score for cervical cancer with squamous histology was 8.5 (range 3–9), indicating moderate to strong and diffuse Trop2 expression. This is in agreement with the results of other studies [3, 22, 29].

Liu et al. [22] reported a significantly higher rate of Trop2 expression in SCC (92.2%) than in adenocarcinomas (79.3%; p = 0.0023). Within the tissue microarray study (TMA) of Zeybek et al. [30] 95% of SCC and 81% of cervical adenocarcinomas stained positive for Trop2 with strong and diffuse expression in 71% and 56% of the tumors, respectively. Using whole tumor sections, SCC showed a significantly higher frequency of Trop2 staining than adenocarcinoma (96.9% vs. 64.1%; p < 0.001) [29]. In the present study, adenocarcinomas showed an insignificantly lower Trop2 expression (see Table 3; Figures 3A,B). This is consistent with the recent results of Mallmann et al. [3].

Data regarding the correlation between Trop2 expression and clinicopathological factors are conflicting. Available data suggest no correlation to patient age and tumor size [22, 29]. Liu et al. [22] reported that Trop2 positivity correlated with lymphovascular space involvement, pelvic lymph node status and FIGO-stage. Those findings are not supported by the results of the most recent studies by Chiba et al. [29] and Mallmann et al. [3]. In the present study, there were no differences in Trop2 expression levels (different IRS scores) within the postsurgical tumor stage and pelvic lymph node involvement (Table 4). This is because all the cases in the present study showed Trop2 immunostaining, and in the majority of cases the expression was moderate to strong (Figures 3A,B).

Liu et al. [22] suggested that high Trop2 expression in cervical cancer cell lines facilitates their escape from the surveillance systems. Therefore, we evaluated Trop2 expression in correlation with peritumoral desmoplastic reaction and lymphocytic response, which are reported to represent features of peritumoral remodelling associated with tumor aggressiveness [39, 55] and tumor control [40, 56, 57]. In the present study, there were no differences in Trop2 expression levels (IRS-scores) when tumors with and without desmoplastic reaction and inflammatory response were compared (Table 4). In contrast, Chiba et al. [29] reported an increased inflammatory response in cervical cancer samples associated with higher Trop2 expression levels. The differences between both studies were caused by the evaluation of different parameters of the inflammatory response (peritumoral lymphocytes and intratumoral CD8/CD3+ lymphocytes) and the incorporation of different histological subtypes within the different study cohorts. Recently, there has been evidence that antibody-drug conjugates may increase the efficacy of immune checkpoint inhibitory therapy [58]. Again, Chiba et al. [29] also found a positive correlation between Trop2 scores and PD-L1 in their study concerning cervical cancers. Nevertheless, further research is needed to compare Trop2 expression and its correlation with immune response features of cervical cancer, e.g., intratumoral versus peritumoral/stromal infiltrating lymphocytes, lymphocytic subpopulations, and immunoregulatory proteins such as PD-L1.

Recent results have reported a decreased prognostic outcome in HPV-negative cervical carcinomas, indicating a stronger prognostic impact of HPV status in tumors with adenocarcinomatous histology [45, 46, 59]. A review by Bujnak et al. [18] compared the effects and adverse events of ADCs from a total of 15 studies on different ADCs in gynecologic oncology. Several trials investigate TRop2-directed ADCs in various gynecologic cancers (e.g., the TROPION-PanTumor03 study [60]), but there is only one trial that deals with the expression of Trop2 in cervical cancers [29]. To our knowledge, however, there have been no studies on the association between Trop2 and p16 expression to date. Immunohistochemical overexpression of the p16 protein is an accepted surrogate marker for high-risk HPV infection in this setting, with a very high concordance with the intratumoral presence of high-risk HPV DNA [61]. Therefore, there may be an unmet need for additional targeted treatment options for patients with HPV-negative (p16-) cervical carcinomas. To the best of our knowledge, no studies have examined Trop2 expression in correlation with p16 status in cervical carcinomas so far. Although only a limited number of cases with p16-negativity was included in the present study (see Table 2; Figures 3A,B), there was no significant difference in Trop2 expression in correlation with p16-immunostaining. Chiba et al. [29] reported low Trop2 positivity (38.5%; 5/13) in gastric type cervical adenocarcinoma, a known HPV-independent subtype of cervical adenocarcinoma [62]. Chiba et al. [29] did not evaluate Trop2 expression depending on HPV-status/p16 expression in detail. In the study of Dum et al. [24], Trop2 expression did not correlate with HPV status in vulvar cancer. Within the studies of Condic et al. [27] and Hoehn et al. [28] HPV-associated vulvar cancer showed predominantly strong and diffuse Trop2 expression in contrast to HPV-independent vulvar cancers.

According to the previously published and present data, Trop2-directed ADC may represent a therapeutic option in cervical cancer patients regardless of histopathological subtype and p16 expression status acting as an immunohistochemical surrogate marker for HPV high-risk association.

Treatment with ADC improves patient outcomes, but acquired resistance may affect treatment results. Previous studies suggest that Trop2 expression levels are likely to influence SG efficacy in triple negative breast cancer patients *in vivo* [35]. A recent *in vitro* study in bladder cancer cell lines showed that loss of Trop2 expression leads to SG resistance [63]. The specific mechanisms of resistance to ADCs (e.g., SG) remain to be investigated in detail [64, 65]. Mechanisms of resistance discussed include acquired reduction of ADC target expression on the tumor cell surface, altered intracellular trafficking, impairment of lysosomal function, drug efflux through efflux pumps, activation of alternative signaling pathways, epigenetic modification/silencing and loss-of-function mutations [64–66]. Preliminary rapid autopsy results from SG-resistant breast cancer patients suggest two main pathways of resistance [66]. Acquired mutation of the payload target (e.g., missense mutation *TOP1E418K*, encoding the SN-38 drug target topoisomerase in SG) leads to SG resistance. Another mutation site may affect the ADC-directed antibody. The acquired missense mutation *TROP2T256R* alters the expression of Trop2 as a target for SG [66]. The *TROP2T256R* mutation results in retained expression of Trop2 in tumor cells, but encodes a protein with altered subcellular localization of the protein: cytoplasmic rather than membrane Trop2 staining [66]. This raises the question of predictive spatial immunohistochemical staining analyses for Trop2 expression on tumor tissue samples. Preliminary results suggest that retained membranous staining is important for SG efficacy, whereas cytoplasmic staining may be an indicator of resistance [66, 67]. Therefore, further research is needed in this context of detailed, pattern based analyses of immunohistochemical staining results. In the present study, no staining pattern of Trop2 restricted to the cytoplasm of cervical cancer tumor cells was seen.

Trop2-positive cervical cancer cell lines exhibit high sensitivity to hRS7 antibody-dependent cell-mediated cytotoxicity [68]. Evaluation of the treatment effect of cisplatin *in vitro*, using the cervical cancer cell lines Siha and CaSki, Trop2 expression was significantly associated with chemosensitivity [22]. *In vivo* results showed that the overall survival of cervical cancer cell lines at 90 days was improved (p = 0.014) in mice with cervical carcinomas treated with SG [30].

In a preliminary study in patients with cervical cancer, Trop2 overexpression was in concordance with increased sensitivity to platinum-based chemotherapy [69]. The included uterine cervical carcinoma showed stable disease with SG treatment in a basket trial [35].

A limitation of the study may be that all the samples tested were taken from patients with upfront surgery, inoperable locally advanced cases, and those with recurrent disease were not included. Accordingly, none of the samples in this cohort had received neoadjuvant therapy. It would be interesting to investigate whether pre-treatment affects the expression of Trop2 in cervical cancer cells. So far, only a small number of studies have addressed this question. For example, Omori et al. hypothesised in their study [70] that neoadjuvant therapies can lead to a change in Trop2 expression in certain lung cancer patients, while research in triple-negative breast cancers showed no significant change in Trop2 expression in non-pretreated vs. pretreated tumours [71].

A further limitation may be the sample size, especially the group of adenocarcinomas (n = 25), so that our results allow only limited general statements on the association between Trop2 and p16 expression. However, they offer an optimistic approach for further studies on larger case groups, including the integration of further methodological considerations (e.g., AI in digital pathology) to strengthen the statements regarding the expression status on tumor cells and to further enable statements on response and resistance.

Regardless of these limitations, the present study indicates high levels of Trop2 expression in cervical carcinomas regardless of their histologic tumor type (squamous versus adenocarcinoma) and p16 expression status. The results of the present and previous studies [3, 29, 30] suggest that SG and other Trop2 ADCs may represent a novel treatment option, including patients with p16-negative cervical adenocarcinomas, and should be explored in clinical trials. Given the promising treatment results with checkpoint inhibitors in cervical carcinomas [14–16] and the positive correlation of Trop2 positivity with immunohistochemical PD-L1 expression and the presence of a high rate of tumor infiltrating lymphocytes (TIL) [29], a combination of Trop2-targeted therapy with immuncheckpoint inhibition is suggested.

## Conclusion

Regardless of the histologic tumor type and p16 expression status, cervical carcinomas show high Trop2 expression and, therefore, Trop2 directed ADCs such as Sacituzumab-Govitecan may represent a promising therapeutic target in this cancer type, including the prognostically poor HPV-independent (p16 negative) tumors, which occur much more frequently in the subgroup of cervical adenocarcinomas than in squamous cell carcinomas.

## Data Availability

The raw data supporting the conclusions of this article will be made available by the authors, without undue reservation.
